# SOCIO-DETERMINANTS OF FRAILTY: HAVE THEY CHANGED OVER TIME?

**DOI:** 10.1093/geroni/igz038.833

**Published:** 2019-11-08

**Authors:** Fiona Matthews, andria Mousa, Carol Jagger

**Affiliations:** 1 Newcastle University, Newcastle, United Kingdom; 2 Department of Infectious Diseases, London, England, United Kingdom; 3 Newcastle University, Newcastle, England, United Kingdom

## Abstract

Socioeconomic inequalities are important drivers of negative health outcomes. This study investigates the effect of social determinants on health using frailty from two studies 20-year apart and examines whether socio-economic differences are widening. A 30-item deficit accumulation score from the baseline data from the two Cognitive Function and Ageing Studies, 1991 (n=7,635) and 2011 (n=7,762) was calculated. For each of the two cohorts separately, binomial regression investigated the relationship between frailty and social determinants. Deprivation was most strongly associated with frailty among the other social determinants. The effect of high deprivation, when compared the lowest tertile, increased in CFAS II (CFAS I: RR=1.21, 95%CI:1.11-1.31; CFAS II: RR=1.47(95%CI: 1.36-1.59). Inequalities in frailty have increased over time, particularly in terms of deprivation. This has important implications for health policy as focusing policies including specific interventions and resources in more deprived areas could greatly improve health outcomes for those areas.

